# Rotating machinery fault diagnosis based on a novel lightweight convolutional neural network

**DOI:** 10.1371/journal.pone.0256287

**Published:** 2021-08-26

**Authors:** Jing Yan, Tingliang Liu, Xinyu Ye, Qianzhen Jing, Yuannan Dai

**Affiliations:** State Key Laboratory of Electrical Insulation and Power Equipment, Xi’an Jiaotong University, Xi’an, China; Fuzhou University, CHINA

## Abstract

The advancement of Industry 4.0 and Industrial Internet of Things (IIoT) has laid more emphasis on reducing the parameter amount and storage space of the model in addition to the automatic and accurate fault diagnosis. In this case, this paper proposes a lightweight convolutional neural network (LCNN) method for intelligent fault diagnosis of rotating machinery, which can largely satisfy the need of less parameter amount and storage space as well as high accuracy. First, light-weight convolution blocks are constructed through basic elements such as spatial separable convolutions with the aim to effectively reduce model parameters. Secondly, the LCNN model for the intelligent fault diagnosis is constructed via lightweight convolution blocks instead of the tradi-tional convolution operation. Finally, to address the “black box” problem, the entire network is visualized through Tensorboard and t-distribution stochastic neighbor embedding. The results demonstrate that when the number of lightweight convolutional blocks reaches 6, the diagnosis accuracy of the LCNN model exceeds 99.9%. And the proposed model has become the most robust with parameters significantly decreasing. Furthermore, the proposed LCNN model has realized accurate, automatic, and robust fault diagnosis of rotating machinery, which makes it more suitable for deployment under the IIoT context.

## Introduction

In view of the significant role bearings play in rotating machinery, it is of the great essence to secure the safe and reliable operations of the bearings. Otherwise, once faults occur, it will not only cause economic loss to the entire industrial system, but also threaten the life of operators. Therefore, it is of great significance to automatically and accurately monitor the state of rotating machinery and take necessary measures in the early stages of failure to ensure the safety of the entire industrial system [1,2].

With the advancement of online monitoring and fault diagnosis technology for rotating machinery, a series of fault diagnosis methods have been introduced to fault diagnosis of rotating machinery in the past half century [3,4]. Traditional fault diagnosis methods can be mainly divided into the construction of fault features and the use of pattern recognition methods for fault classification. As for fault feature construction, signal processing methods, such as wavelet transform, wavelet packet transform, empirical mode decomposition as well as variational mode decomposition, are adopted to construct the fault characteristic parameters. Then dimensionality reduction is realized through principal component analysis and auto-encoder of the constructed parameters [5–8]. The final key feature parameters are selected in this process. As for feature-based pattern classification methods, they include support vector machines, decision trees, random forests, and artificial neural networks [9,10]. As narrow models, however, the traditional fault diagnosis methods find it in no position to perfectly represent the nonlinear relationship between fault signals and fault categories. Moreover, they to a large degree rely on signal analysis and processing methods and diagnostic experience [11,12].

Confronted with the massive acquired data brought about by the development of Industry 4.0 and the Industrial Internet of Things (IIoT), deep learning has emerged as a multi-level model for fault diagnosis of rotating machinery, for it can well represent the non-linear relationships of faults. In particular, deep belief networks and auto-encoders are typical representative [13–15]. F. J. et al. proposed a deep neural network-based fault diagnosis method for rotating machinery. Through adaptively mining fault features from the frequency spectrum, it can be used for the diagnosis of various problems and in the meantime effectively classify whether the machines are on health condition [16]. Z. C. et al. constructed a SAE and DBN-based multi-sensor feature fusion method for bearing fault diagnosis and verified its high recognition rate and low sensitivity to training samples [17]. S. H. et al. adopted a deep auto-encoding feature learning method for rotating machinery fault diagnosis, which demonstrated better robustness and effectiveness in feature learning and fault diagnosis [18]. C. L. et al. used superimposed denoising auto-encoder (SDA) for the health status recognition of signals in the context of environmental noise and fluctuations in working conditions, which had high accuracy and strong robustness [19].

With significant automatic feature extraction capability, convolutional neural network (CNN) has achieved excellent fault diagnosis performance as an important branch of deep learning. S. G. et al. used a CNN model to directly classify a continuous wavelet transform image (CWTS) for diagnosis with no need for dimensionality reduction to avoid information loss [20]. W. Y. et al. designed a broad convolutional neural network (BCNN) with incremental learning ability to perfectly capture the fault process features. Moreover, it can effectively realize self-update to include new abnormal samples and fault classes. J. J. et al. proposed a deep coupled dense convolutional network (DCDCN) that well combined data fusion, feature extraction and fault classification together for intelligent diagnosis. As for the multi-scale feature learning of complex vibration signals. G. J. et al. developed a new systematic structure of multi-scale feature learning-based wavelet transform gearbox for intelligent fault diagnosis. By introducing coarse-grained layers to traditional CNNs, the integration of multi-scale feature learning was realized [21]. G. X. et al. proposed an online fault diagnosis method based on the deep transfer convolutional neural network (DTCNN). After directly transferring the shallow layers of the trained offline CNNs to the online CNNs, the online CNNs can significantly improve the real-time performance, and the diagnostic accuracy problem was successfully solved in limited training time [22].

In order to improve the fault diagnosis accuracy, the main method is to deepen the depth of the model. However, limited by the sample size, as the model deepens, the CNN may encounter the overfitting problem or even the vanishing gradient. Although the above problems can be solved by transfer learning, transfer learning fails to take account of the parameter number and storage space of the model, and in the meantime, it requires similar samples for the migration objects and the problems to be solved. In order to avoid severe overfitting and vanishing gradient in the process of model deepening, as well as effectively reduce the model parameters and storage space, in this paper a lightweight convolutional neural network (LCNN) is constructed for automatic and accurate intelligent fault diagnosis of rotating machinery via lightweight convolution blocks instead of traditional convolution operations. The specific contributions are as follows:

1. The LCNN model was constructed via a lightweight convolution block rather than a traditional convolution operation, which greatly improves failure diagnosis accuracy and significantly reduces computational complexity and model memory. In addition, strictly speaking, it solves the serious overshoot and bending gradient problems caused by the deepening of the model in a limited number of samples when the accuracy is high.
2. Through constructing different LCNN models with multiple lightweight convolutional block numbers, the impact of network depth on the performance of fault diagnosis of rotating machinery is studied. When the number of blocks reaches a certain extent, the recognition accuracy will gradually remain stable, but the model parameters will increase exponentially. By comprehensively considering the model accuracy and parameter storage, the optimal fault diagnosis model for rotating machinery can be determined.
3. In order to give full play to the powerful feature extraction advantages of convolution, we propose to use multiple convolution sizes instead of a single convolution size during the construction of the convolution block. A large-scale convolution kernel is used to extract fault representation features more effectively, thereby improving the recognition accuracy of the model.

## Basic theory

### CNN

As a deep learning model for image recognition, CNN is mainly composed of input layer, convolutional layer, pooling layer and fully connected layer. With the further development of the CNN model, the convolutional layer not only includes convolution operations, but also includes batch normalization and nonlinear activation. Currently, it is typical of the CNN model to own all three types of operations in the convolutional layer [23].

The input data of the CNN model is generally the original image X. In this paper, Z_i_ refers to the output characteristics of the l-th layer of the convolutional layer in the model, where Z_0_ = X. And then the generation process of Z_i_ can be described as Eq (1), where W_i_ refers to the weight vector of the convolution kernel of the l-th layer [24]. After convolution operation with the output feature data in the l-1_th layer, it is further added to the offset vector b_i_ in the l-th layer. And then a non-linear activation operation is performed on the additive result via f(*x*) function to obtain the output feature Z_i_ in the l-th layer. After the convolutional layer, there is usually a pooling layer. This layer can reduce the dimensions of features while retaining spatial information. Finally, the fully connected layers are used to map the abstract feature information extracted by all previous layers to the sample marker space during the training process to the sample marker space. After training, the probability distribution Y of the original input image data can be acquired, as shown in Eq (2).


$$Z_{l}=f(Z_{l‐1}*W_{l}+b_{l})$$ (1)

$$Y_{i}=P(L=l_{i}|X;(W,\;b))$$ (2)

$$\mathrm{MSE}=\frac{1}{n}\sum_{i}^{n}{\left({\hat{Y}}_{i}‐Y_{i}\right)}^{2}$$ (3)

$$W_{i}=W_{i}‐\eta \frac{\partial L(W,\;b)}{\partial W_{i}}$$ (4)

$$b_{i}=b_{i}‐\eta \frac{\partial L(W,\;b)}{\partial b_{i}}$$ (5)

The mathematical characteristic of CNN is to perform several linear and non-linear transformations on the matrix Z_0_ of the initial input image, and finally map it to another new dimensional space Y. The training process of the CNN model is to minimize the cost function L(W, b) of the model. In other words, this is a process of minimizing the gap between the predicted result obtained by the inference operation of the input image matrix Z_0_ and the expected result. The commonly used cost equations include cross entropy and mean square error (MSE) equations, where MSE is shown in Eq (3). In addition, as shown in formulas (4) and (5), the gradient descent algorithm is widely used to optimize the cost formula. The gradient descent method propagates the loss value of the model and updates the parameters in the model layer by layer, where η represents the speed of learning and parameter update. A lot of research on CNN helps to significantly improve the basic network structure, especially the depth of the model. With the deepening of the model, more feature information can be extracted, thereby improving the recognition accuracy

### Spatial separable convolutions

Despite the good feature extraction effects in data processing, traditional CNNs cannot be applied to devices with limited hardware performance due to high computational complexity. In order to improve the above disadvantage, a LCNN model is constructed via the lightweight convolutional operations instead of the traditional convolutional operations, which is capable of effectively improving the accuracy as well as reducing the calculation and storage cost [24,25]. Among the lightweight convolution operations, including depth-wise separable convolutions, channel shuffling, and spatial separable convolutions. Spatial separable convolutions can promote the faster learning without losing feature information as much as possible after being applied through spatial aggregation over deep separable convolutions [26].

As for depthwise separable convolution, its convolution process includes the operations of deep convolution operation layers and pointwise convolutions. Assume that the size of the input data is D_f_×*D*_f_×M, where M is the number of feature channels of the input data. The deep separable convolution first uses M convolution kernels of size D_k_×*D*_k_×1 for deep convolution. Each convolution kernel performs convolution operation on only one input feature channel. In this way, M feature channels can be obtained after these M convolution kernel operations. Under reasonable settings, the output data is D_f_×*D*_f_×M, and the calculation amount is D_k_×D_k_×*D*_f_×*D*_f_×M. Then, the output results of the deep convolution layer are subjected to pointwise convolution operations through N convolution kernels with a size of 1×1×M. The final output data size is D_f_×D_f_×N. The calculation amount is M×D_f_×D_f_×N. After the above decomposition, the ratio between the ultimate calculation amount and the calculation amount the original standard convolution requires is shown in Eq (6). As can be obtained from Table 1, the ratio of the calculation amount of spatially separable convolution and traditional convolution is:

$$\frac{D_{k}\times D_{k}\times D_{f}\times D_{f}\times M+M\times D_{f}\times D_{f}\times N}{D_{k}\times D_{k}\times M\times D_{f}\times D_{f}\times N}=\frac{1}{N}+\frac{1}{D_{k}^{2}}$$ (6)

**Table 1:** The number of parameters and computation amount for traditional convolution and spatial separable convolution.

	traditional convolution	spatial separable convolutions
Parameter	*D*_*k*_×*D*_*k*_×*M*×*N*	*D*_*k*_×*D*_*k*_×*M*+1×1×*M*×*N*
Calculation amount	*D*_*k*_×*D*_*k*_×*M*×*N*×*D*_*F*_×*D*_*F*_	*D*_*k*_×*D*_*k*_×*M*×*D*_*F*_×*D*_*F*_+*M*×*N*×*D*_*F*_×*D*_*F*_

Considering that *D*_*k*_^2^ is much larger than N, and the ratio of the calculation amount between the two is approximately 1/*N*, it can be seen that the calculation amount of the lightweight convolutional neural network constructed by spatially separable convolution is significantly reduced.

Research shows that the convolution size is proportional to the calculation amount. For example, with the same filter, the calculation amount of 5x5 convolution kernels are 25/9 = 2.78 times larger than that of 3x3 convolution kernels. Compared to the 3x3 convolution kernel, the 5x5 convolution kernel has a broader “field of view”, which can capture more information. Therefore, simply reducing the kernel size will result in information loss. In this sense, a multilayer network is adopted in place of 5x5 convolution. Through regarding a 5x5 network as full convolution, each output is a kernel sliding on the input. Also, it can be replaced by a two-layer 3x3 full convolution network, and the information loss at this time can be ignored.

In fact, it is better for a convolution to be decomposed as asymmetric. For example, a 3x1 convolution, followed by a 1x3 convolution, is equivalent to a 3x3 convolution. The calculation of the two-layer structure is reduced by 33%. If it is replaced by the 2x2 convolution, the calculation declines only by 11%. Theoretically, nxn convolution can be replaced by 1xn -> nx1 convolution. As n increases, it can reduce more calculations. In practice, such decomposition effects are not good in the first few layers of the network while in a medium network, it demonstrates this good performance (for feature map of mxm size, m is between 12 and 20). At these sizes, 1x7 -> 7x1 possesses an excellent convolution effect. The aforementioned decomposition operation mainly deals with the spatial dimensions of the image and the kernel, namely width and height. The operation of this kind is a spatial separable convolution operation.

Spatial aggregation can be accomplished by embedding in lower dimensions without losing much or any representation ability. Before performing a wider convolution, the number of input dimensions can be reduced before spatial aggregation without serious negative effects. Due to the strong correlation between neighboring cells, if the output is used for the spatial aggregation context, much less information is lost during the dimensionality reduction process [27]. Considering that these signals are easily compressed, dimensionality reduction can even promote faster learning.

## Method

### Lightweight convolution blocks

**Fig 1:**
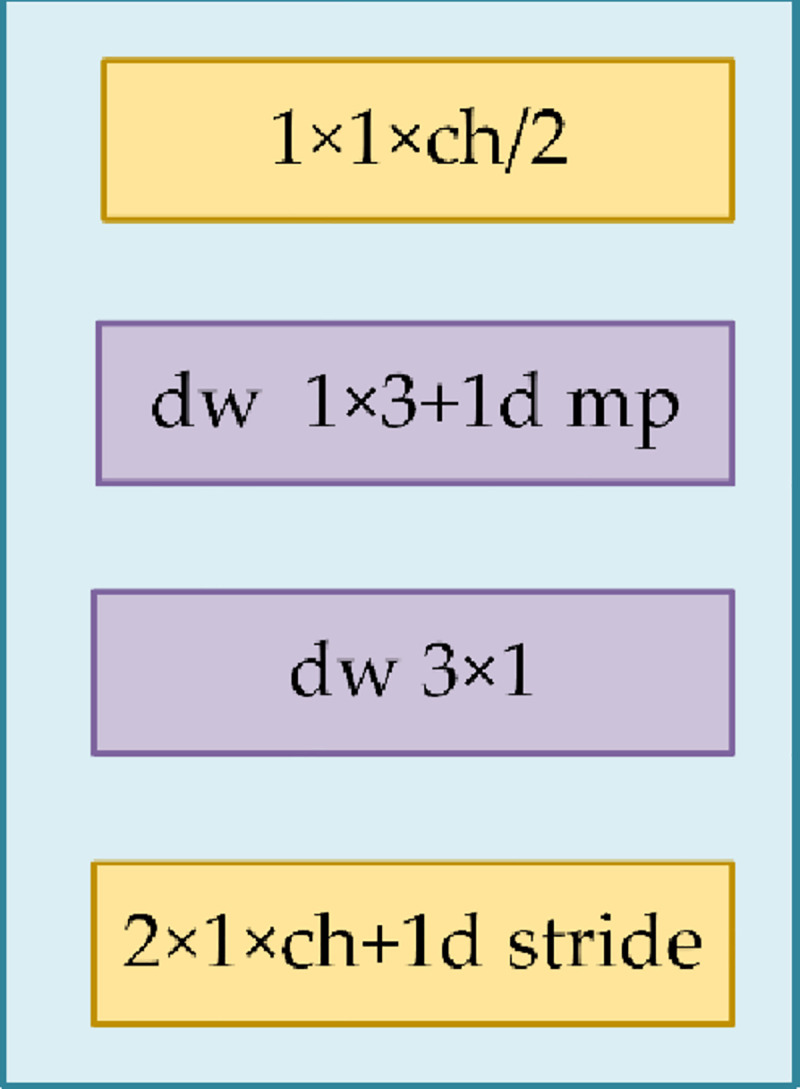
Lightweight convolution block operation diagram.

In order to design LCNN model, a lightweight convolution block with a general structure is designed to seamlessly replace the ordinary convolutional layers in the simplified network. It can largely solve the data compression problem and in the meantime effectively explore the network structure. The lightweight convolution block operation proposed in this paper is shown in Fig 1, where “dw” refers to deep convolution, “mp” refers to max-pooling, and “ch” refers to the number of output channels. Firstly, three deep convolutions are decomposed into two linear layers, which guarantees that pooling processing is performed after the first spatial layer, and thereby saves the calculation results to the second layer. Then, the sub-sampling is segmented along the spatial dimension, and a 1 × 2 maxpooling kernel is applied after the first deep convolution. For the second sub-sampling, the 2×1 kernel and a corresponding step size are chosen to replace the ordinary pointwise convolution.

As one of the cores of ResNet, Bottleneck Architectures greatly reduce the network parameters while effectively simplifying the training process. The Bottleneck structure is to reduce the parameter number. Bottleneck mainly can be divided into three steps: first, reducing the data dimensions, then performing the convolution of the conventional convolution kernels, and finally increasing the data dimensions (similar to the hourglass). According to reference, a quarter of the output channels are no longer used, but a factor of 0.5 is identified, and the minimum channel number is 6.

**Fig 2:**
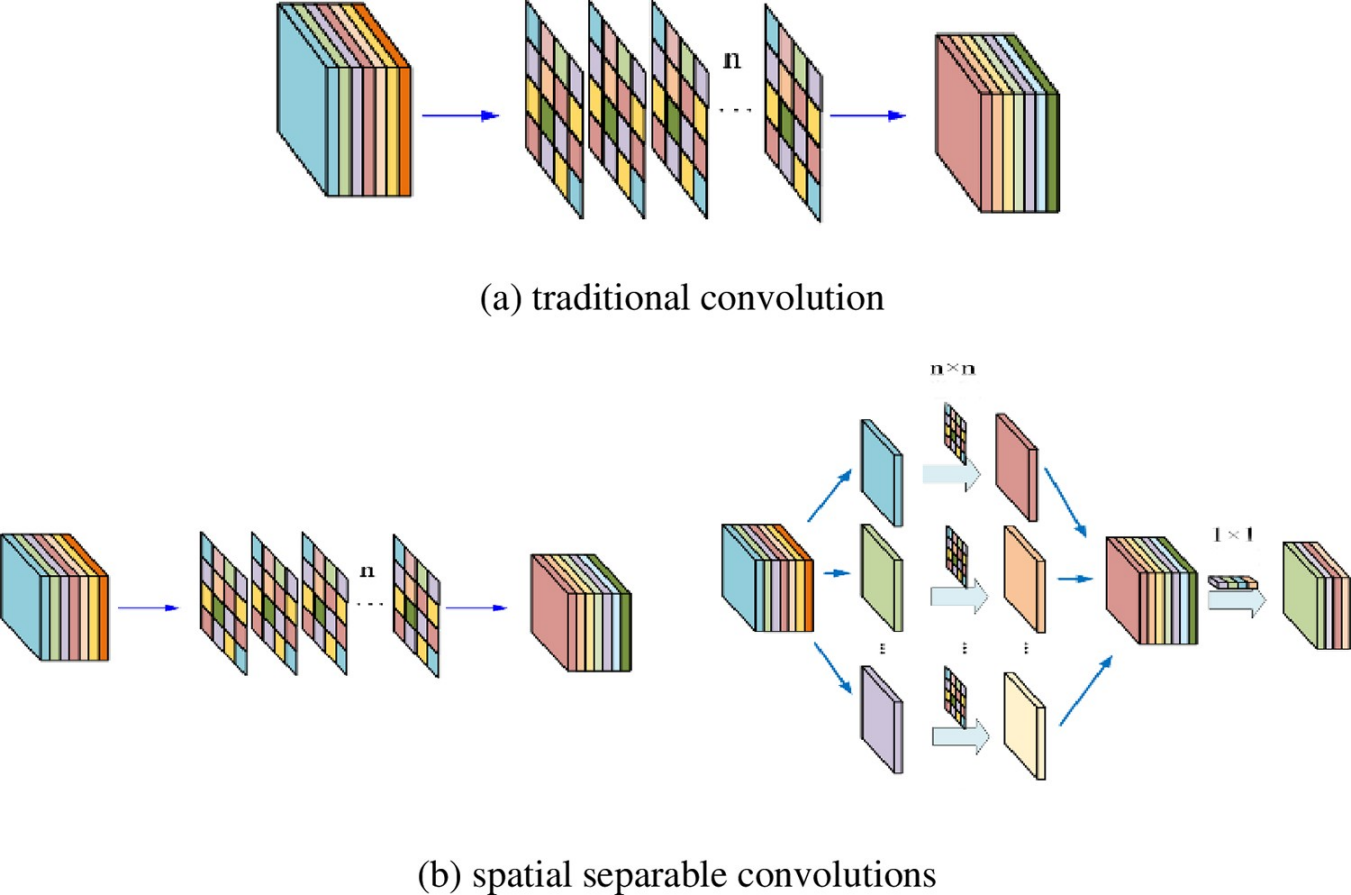
The schematic diagram of traditional convolution and spatial separable convolution. (a) traditional convolution (b) spatial separable convolutions.

As shown in Fig 2, the proposed lightweight convolution block uses spatially separable convolution operations instead of traditional convolution operations, which is very representative. The traditional convolution calculation is to perform convolution on 3 channels at the same time; the spatially separable convolution calculation is divided into two steps: first, convolution is performed on the 3 channels separately and 3 values are output after one convolution, and then a value is obtained through a 1x1x3 convolution kernel.

The proposed lightweight convolutional neural network, as an efficient convolutional neural network designed for mobile terminals, not only solves the problem of data compression, but also reduces computational overhead and storage space while ensuring accuracy. This will enable larger networks to be deployed on low-capacity hardware and improve the efficiency of use of existing hardware.

### Model training

The stochastic gradient descent (SGD) algorithm, namely SGD optimizer, is used for training. During the training process, all training parameters are uniformly maintained and updated by the SGD optimizer, with the same learning rate. When training reaches a certain stage, the loss curve may no longer drop, and the parameter learning becomes difficult. It is necessary to reduce the learning rate so that the model loss continues to decrease during training. When training the target recognition network, SGDR is used to adjust the learning rate.

After completing the training, the pruning method can be adopted to further compress the model volume and operation amount, and improve the running speed. The core task of pruning is to measure the importance of the convolution kernel to the final result. In this paper, we mainly analyze the kernel cut for spatial separable convolutional layers in the network. In addition, the parameters in the Batch Normalization (BN) after the convolution layer are mainly used to measure the importance of the convolution kernel.

The major function of the BN layer is to avoid data shifting among multiple layers. The main method is to renormalize the data distribution to a distribution with a mean of 0 and a variance of 1. In order to maintain the expressive competence of the model, the normalized data is endowed with a bias β and a coefficient α.

In the BN layer, both α and β are training parameters. After training, each BN layer has specific values for corresponding α and β. Since the BN layer exclusively corresponds with the respective convolutional layer, the coefficient α can roughly represent the importance of the corresponding convolution. In this paper, we mainly analyze the coefficient α of BN in each layer or each module to measure the importance.

### Model framework

**Fig 3:**
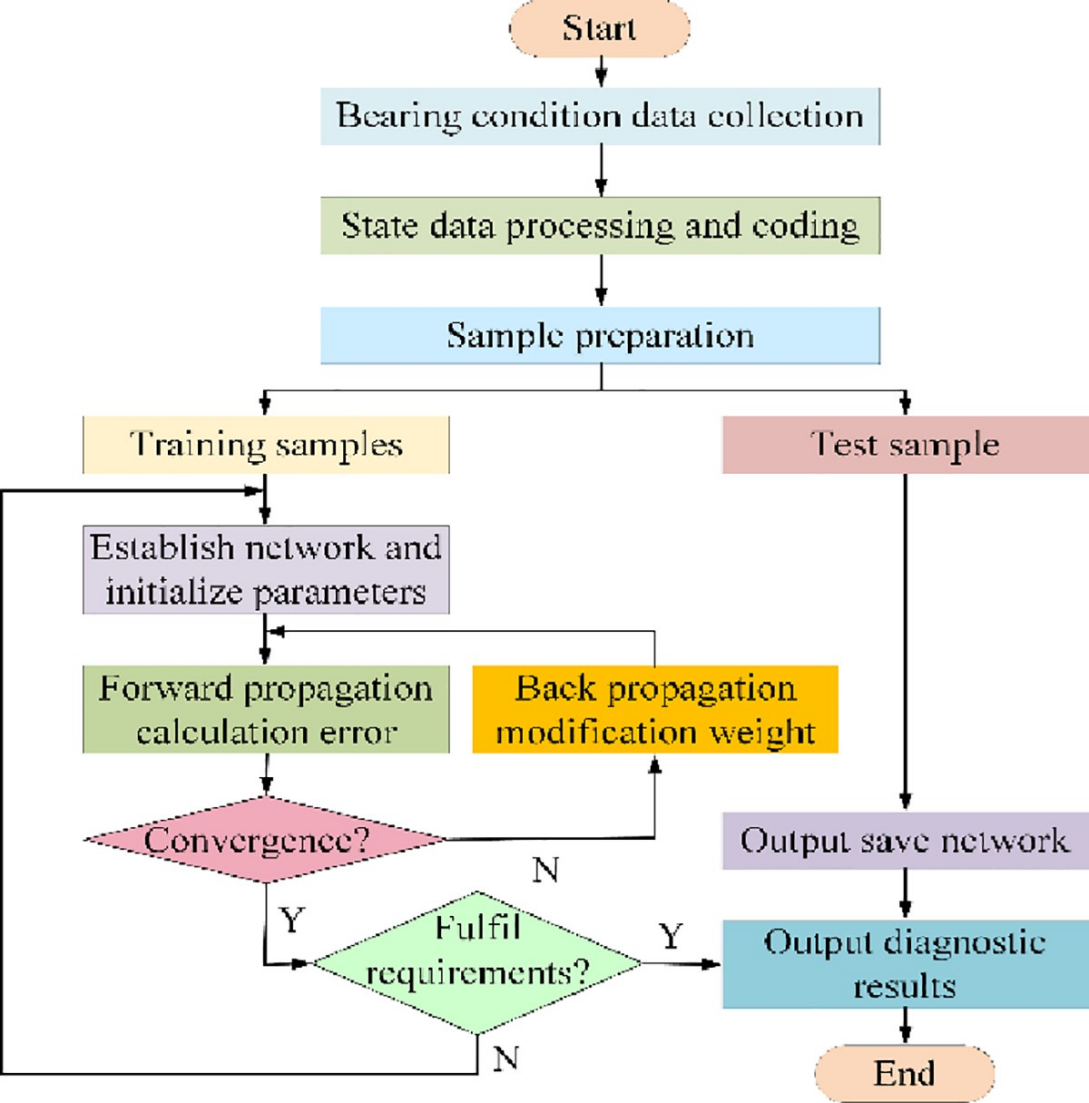
LCNN-based bearing fault diagnosis process.

To sum up, in this paper lightweight convolution blocks are constructed via spatial separable convolution. And the constructed blocks are used to build a novel LCNN model. The entire network construction and diagnosis process is presented in Fig 3. A LCNN-based fault diagnosis framework for bearings is summarized in the following four steps:

1. Construction of lightweight convolution blocks. Through tentative experiments apart from previous studies, spatial separable convolution operation is used to construct lightweight convolution blocks in place of the traditional convolution operations, which effectively compresses the model.
2. Network construction. By using the lightweight convolution blocks constructed above instead of the traditional convolution operations, an effective LCNN is constructed for fault diagnosis.
3. Network training and optimization. The model has been executed at least five times to eliminate the effects of random initialization. During model training, SGD is used as an optimizer, and the pruning method is used to further compress the model volume and operation amount to improve the model running speed.
4. Model deployment. The trained LCNN is extracted to construct the fault diagnosis model. Deploy the fault diagnosis model on bearings for real time monitoring and fault diagnosis.

**Fig 4:**
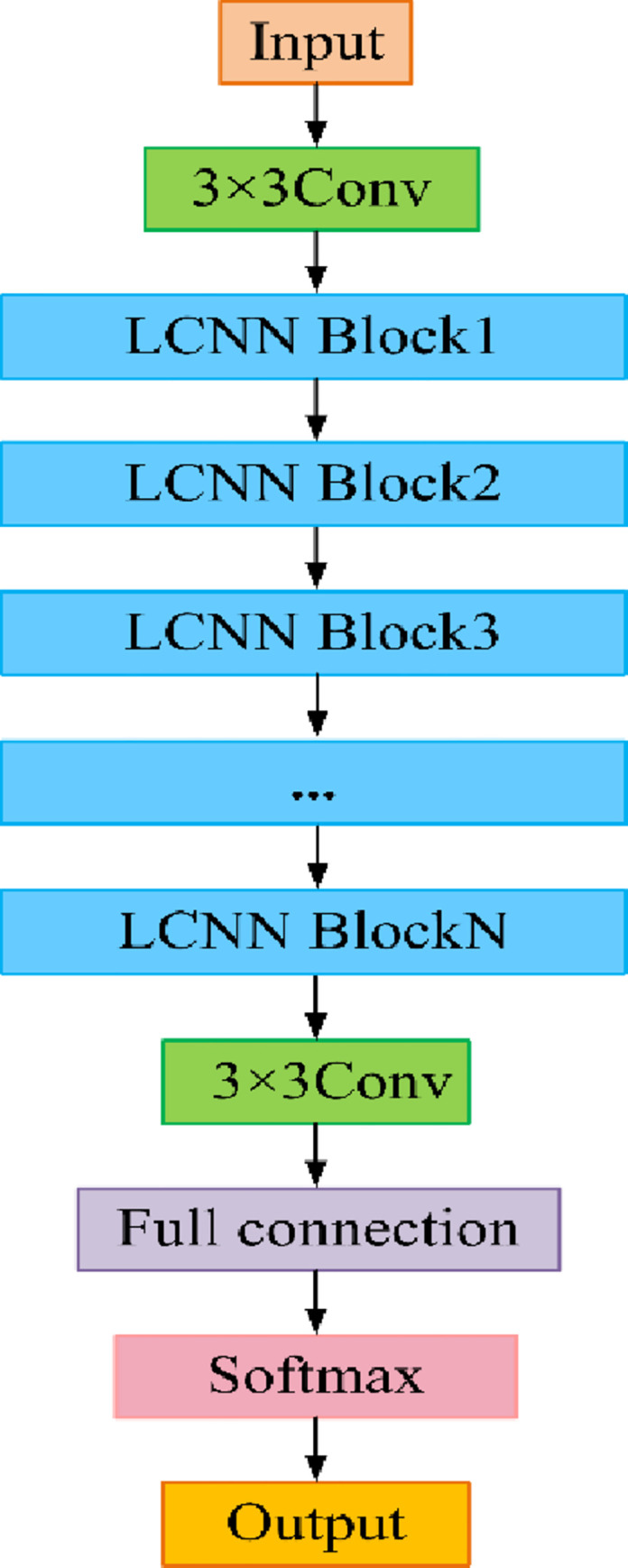
The overall framework of four types LCNN architectures.

The LCNN model is constructed by using LCNN blocks. With little impact on the accuracy, this model retains the content that has been largely reduced in the context of enormous decrease of calculation amount. Besides, this novel convolution module is able to deploy larger networks on low-capacity hardware, or improve the efficiency of existing models. Compared with the traditional narrow models, the CNNs manifest excellent performance in the diagnosis of bearing faults. The increase of model parameters, helps to improve the model’s ability, but increases the overfitting risk with limited supervised training. For credible comparative research, we have designed four types of LCNN architectures with 3–6 EffNet blocks, LCNN overall framework as shown in Fig 4.

To achieve layered and abstract feature representation, the number of kernels increases with depth increasing. The dropout layer is added to avoid overfitting. The final output layer gives the diagnostic category for each sample. The activation function is ReLU, the batch size is 16 to 64, and the learning rate of optimizer is 0.01. At the same time, the results of certain typical DCNN models and stack auto-encoders are used for comparison. The impact of the block numbers on the model is also studied. During the experiment, we have trained our model with Keras (Tensorflow backend) on a machine that has a GeForce RTX 2060 GPU, an Intel i7-8700 CPU and 16 gigabytes of RAM [28].

## Experimental verification

### Results on bearing fault dataset of Western Reserve University

As one of the most influential public datasets for mechanical fault diagnosis, Case Western Reserve University Bearing fault dataset is regarded as a reference for research and verification of the fault diagnosis performance of the proposed model. The test object of the CWRU bearing center data acquisition system is the driving end bearing, and the bearing type is a deep groove ball bearing SKF6205. The faulty bearing is made by electrical discharge machining (EDM), and the sampling frequency is 12 kHz. The bearing has a single-point fault set by the EDM process. The fault diameter includes four sizes, namely 0.178 mm, 0.356 mm, 0.533 mm, and 0.711 mm.

In this paper, the driving end vibration signals are selected as the experimental data, including the vibration signals in 4 different states, namely Normal (NOR), Ball Fault (BF), Outer Race Fault (ORF), and Inner Race Fault (IRF). The signals collected in each state vary because of the different fault diameters and loads. The load size is respectively 0W, 746W, 1492W, and 2238W. The 12k Drive End Bearing Fault Data is diagnosed by using 200 data points at a time. In total, 10 types of signal data are constructed for experiment, which cover the 4 signal states. Reference [19], the 10 types of data are 0.178mm BF (0), 0.178mm IRF (1), 0.178mm ORF (2), 0.356 BF (3), 0.711mm IRF (4), 0.356mm ORF (5), 0.533mm BF (6), 0.533mm IRF (7), 0.533mm ORF (8), NOR (9).

In this section, we focus on the performance of various methods on the Case Western Reserve University Bearing fault dataset. First, the influence of the number of lightweight convolution blocks on the recognition results is explored. Table 2 gives the fault diagnosis results of different block numbers. When the number is 6, the diagnostic accuracy of the model has reached 99.91%. As the number increases further, the model accuracy increases slightly, but the parameter amount increases exponentially, which brings difficulties to model training and deployment.

**Table 2:** The fault diagnosis results of different lightweight convolution block.

Model	Accuracy (%)	Parameter (million)	Storage (MB)
LCNN-6	99.91	7.061	27.845
LCNN-5	99.45	1.853	7.340
LCNN-4	97.94	0.626	2.590
LCNN-3	94.09	0.478	1.975

**Fig 5:**
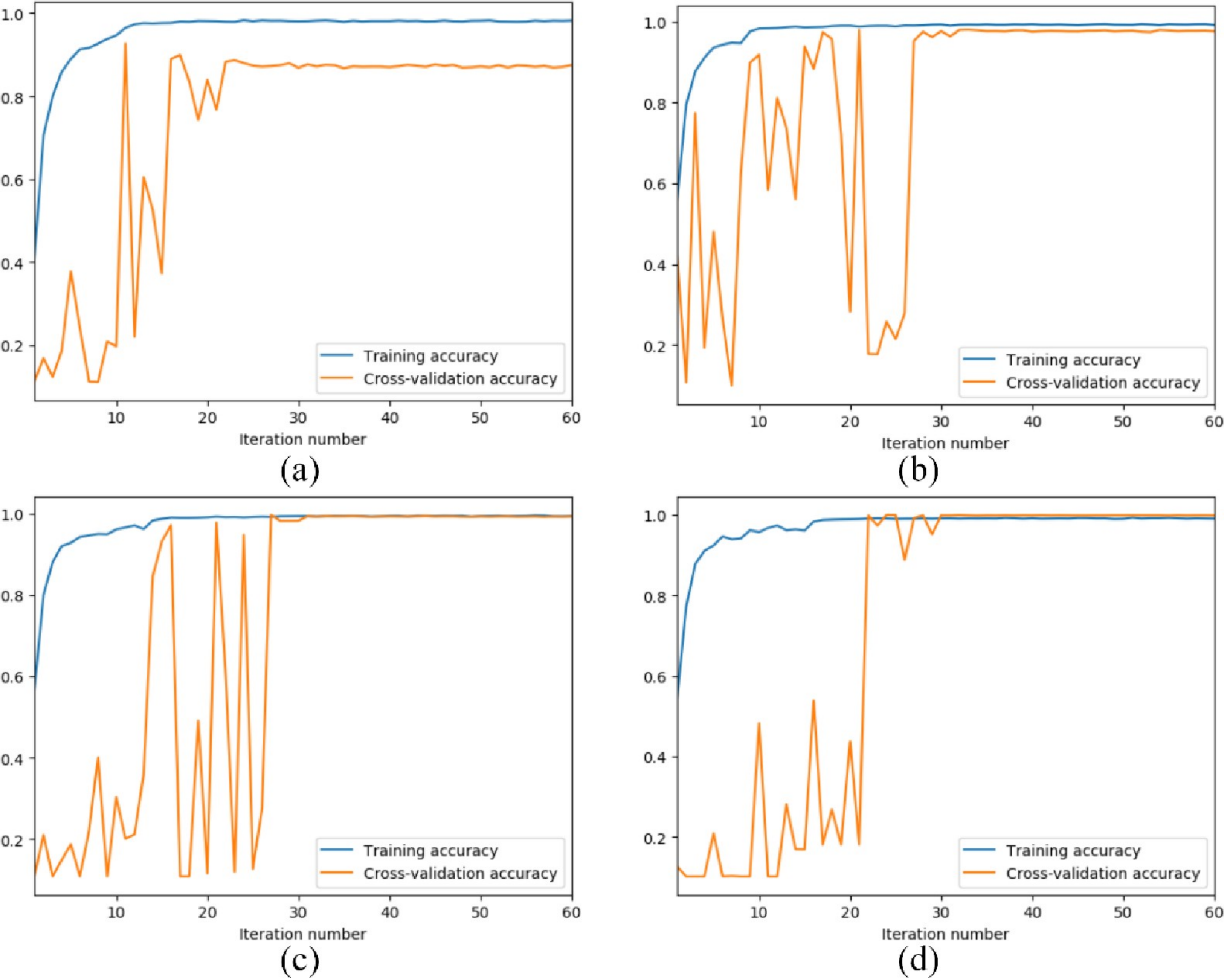
The training curves of the LCNN models constructed by different number of lightweight convolution blocks. (a) 3 blocks. (b) 4 blocks. (c) 5 blocks. (d) 6 blocks.

Fig 5 presents the training accuracy and cross-validation accuracy curves of the models constructed by block numbers. With the block number increasing, the training of LCNN takes fewer iterations to be stable, indicating the robustness and high cross-validation accuracy of the model. In this sense, this paper chooses the LCNN model constructed by six lightweight convolution blocks as the best model for intelligent fault diagnosis of rotating machinery.

**Fig 6:**
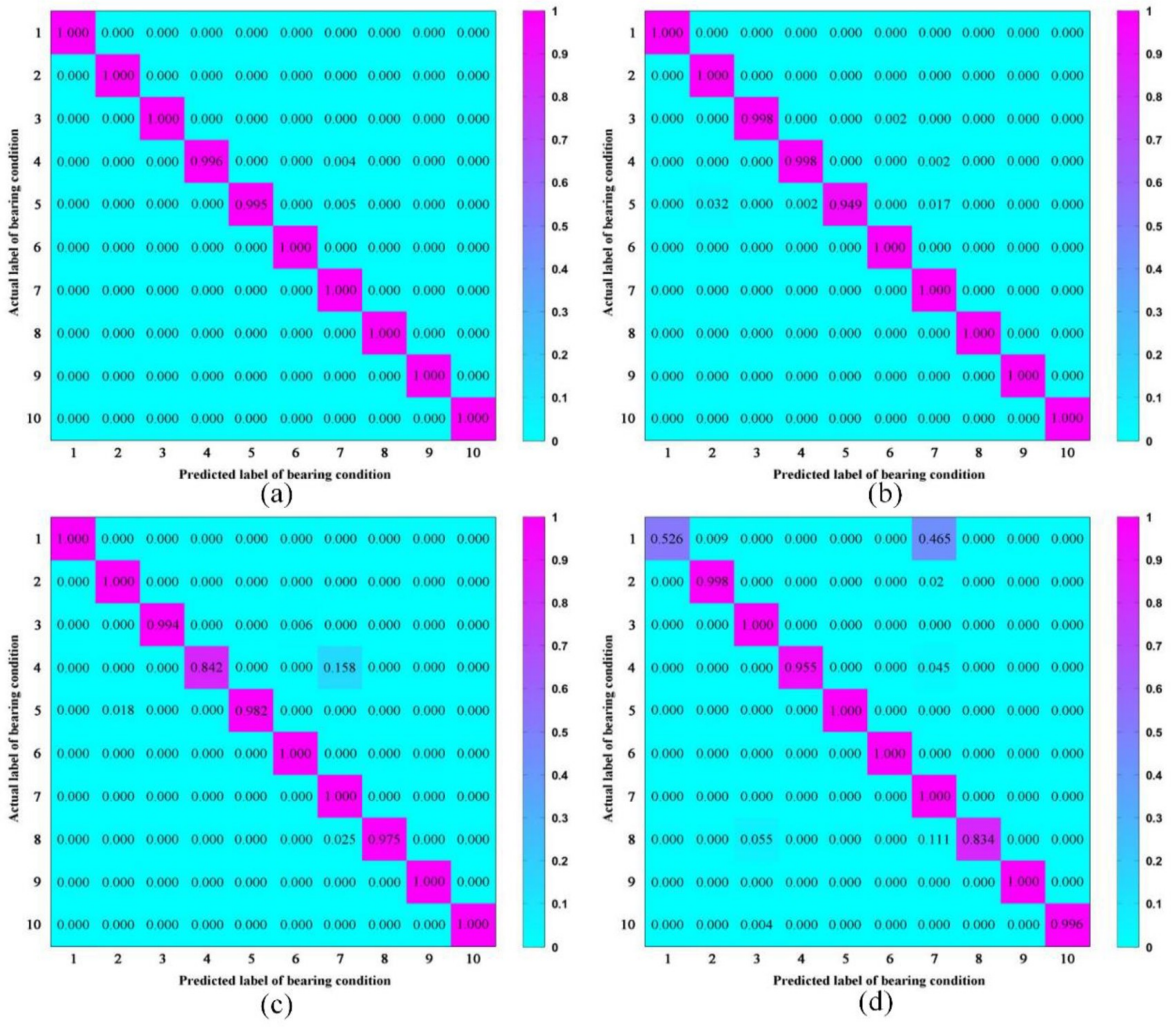
Confusion matrices of the LCNN models with different numbers of lightweight convolutional blocks. (a) 6 blocks. (b) 5 blocks. (c) 4 blocks. (d) 3 blocks.

Fig 6 shows the confusion matrix results of the LCNN models with 4 different block numbers. From the confusion matrix, LCNN-3 has the lowest diagnostic accuracy on data type (0), while the other LCNN models have low accuracy on type (3) and (4), and demonstrate high accuracy on type (6). The confusion matrix results further prove that LCNN model with 6 lightweight convolutional blocks possesses the best accuracy performance.

Table 3 demonstrates the fault diagnosis results of the proposed method and the traditional methods. The proposed LCNN model significantly outperforms in recognition accuracy. Limited by the data sample size, the LeNet and AlexNet models suffer severe overfitting or even the vanishing gradient problem in the training process. Therefore, migration training is used to achieve high accuracy.

**Table 3:** PD recognition results of different methods.

Model	LCNN	LeNet	AlexNet	ResNet	DBN	SAE	SVM
Results(%)	99.91	99.56	99.63	87.96	87.20	92.15	87.88

**Fig 7:**
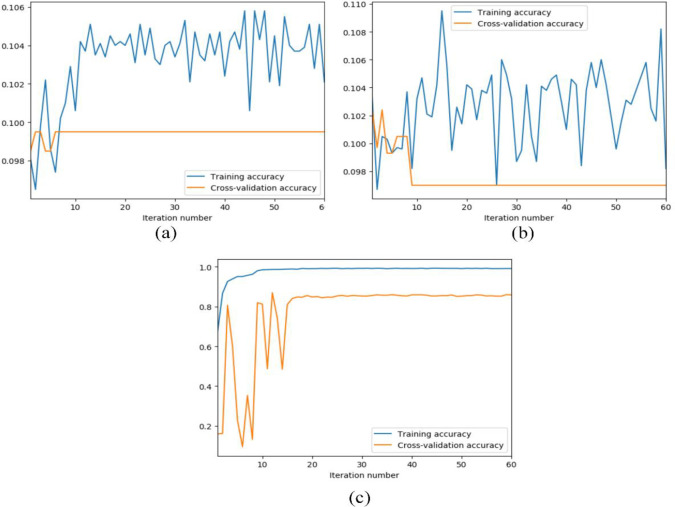
The training and testing curves of the CNN models. (a) LeNet. (b) AlexNet. (c) ResNet.

Table 4 presents the parameters and weight storage of the proposed LCNN model and traditional CNN models. It is evident that the proposed LCNN demonstrates much more advantages with significantly less parameters and storage. Fig 7 shows the training and testing curves of the CNN models. Obviously, LeNet and AlexNet have suffered the overfitting and even vanishing gradient problem owing to the limited sample size.

**Table 4:** The compare results of the LCNN and traditional CNN models.

Index	Model	Parameter (Million)	Weight storage (MB)
1	LCNN	3.06	12.522
2	ResNet	11.18	43.647
3	LeNet	14.43	56.396
4	AlexNet	24.72	96.695

**Fig 8:**
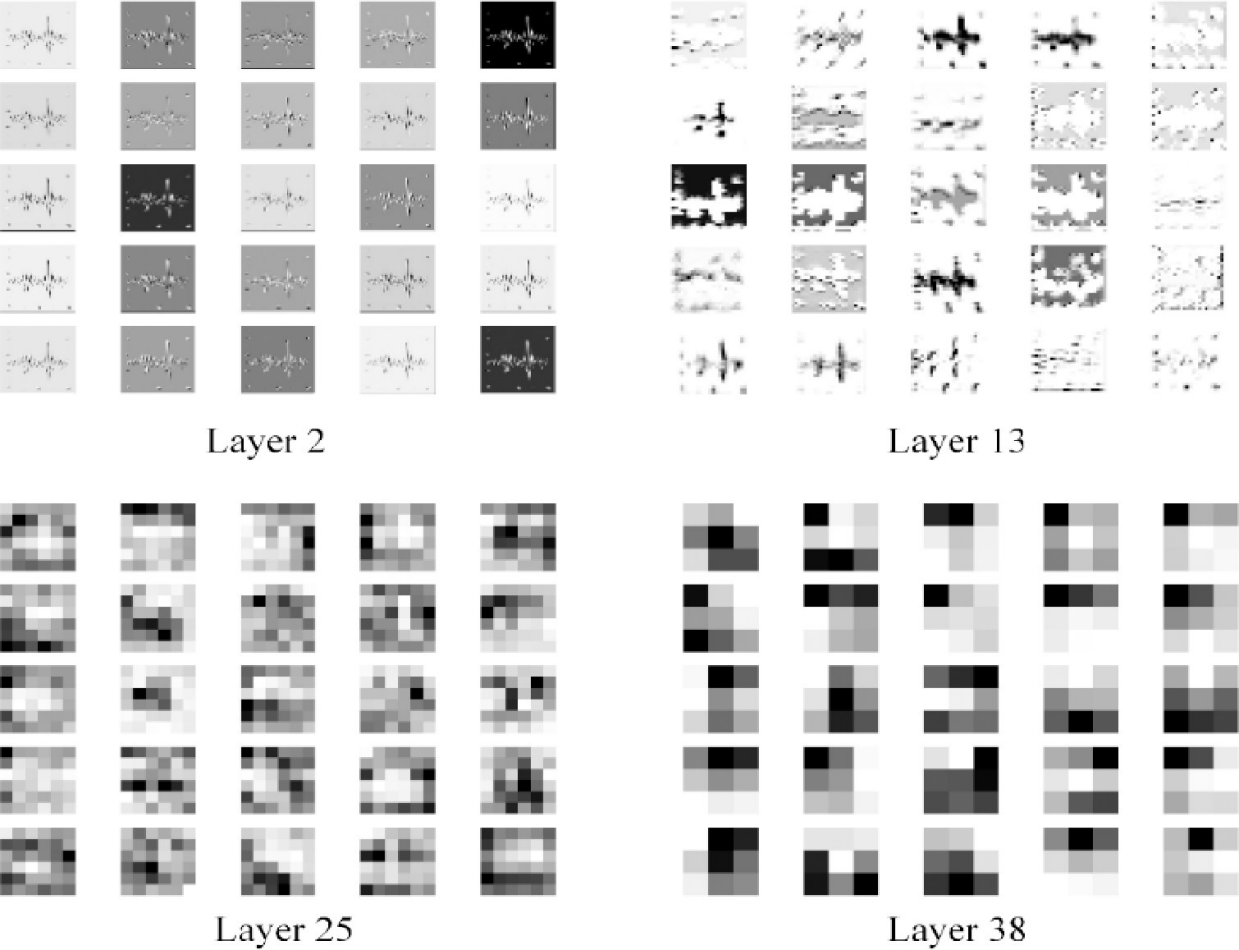
The feature map of each convolutional layer of the LCNN model. (a) Layer 2. (b) Layer 13. (c) Layer 25. (d) Layer 38.

Since the CNNs are usually called as “black box”, TensorBoard is adopted to visualize the extracted features of each convolutional layer of the LCNN model proposed in this paper. The feature map is shown in Fig 8. Initially, the convolution mainly extracts edge features, and then becomes increasingly abstract. And the feature map becomes growingly smooth, indicating that the feature representation is sufficient.

**Fig 9:**
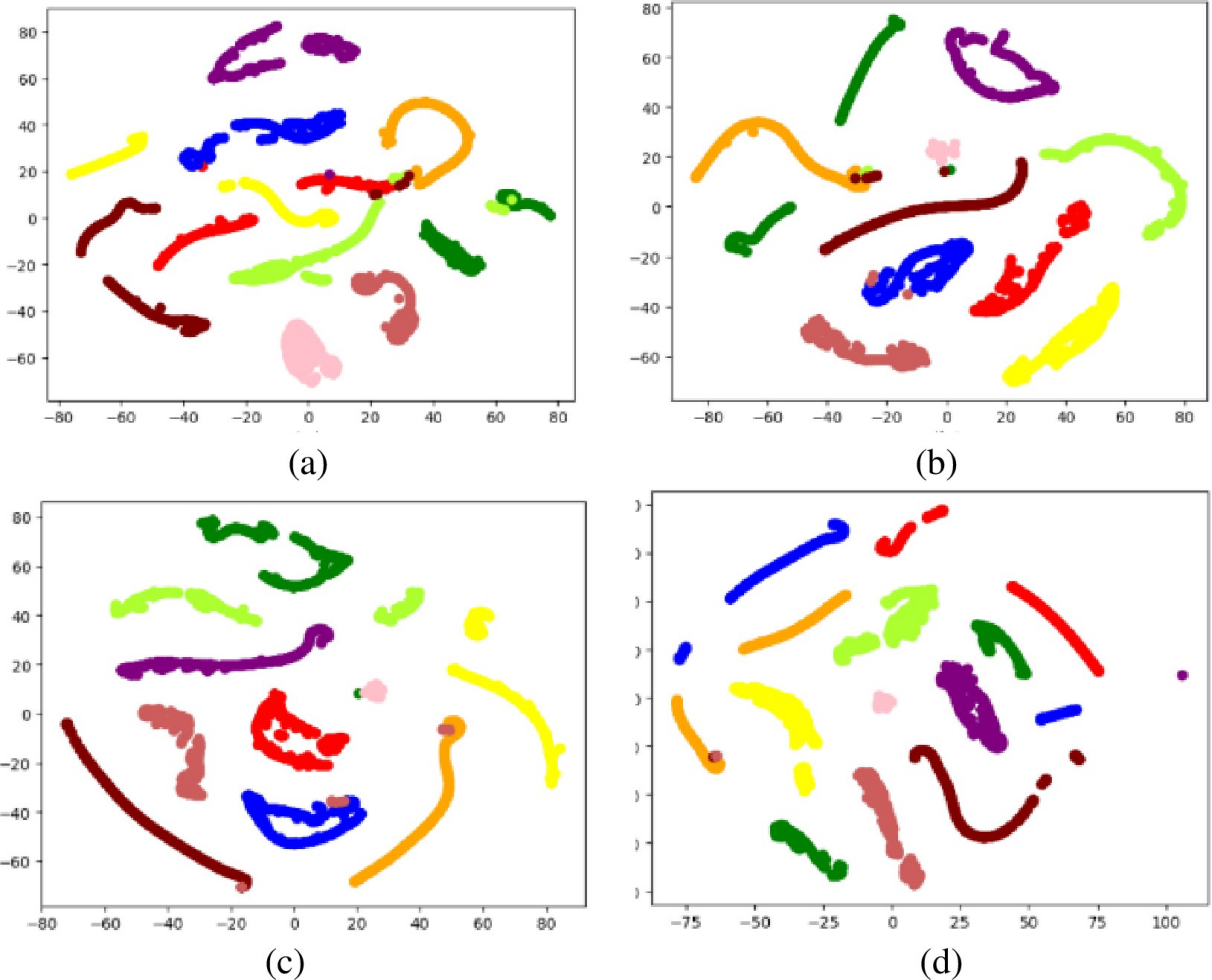
The visualization results of t-SNE. (a) LeNet. (b) AlexNet. (c) ResNet. (d) LCNN.

In order to provide an intuitive explanation of the model prediction, t-SNE is used to visualize the learned features in a hidden fully connected layer [29,30]. The visualization results are shown in Fig 9. In the flat hidden fully connected layer, the samples under the same fault condition are clearly collected together and even separated, which indicates that the learned feature descriptors have good feature representation capabilities [31–33]. After performing non-linear mapping in the classifier, the features under different fault conditions are well separated in the last hidden fully connected layer, except for slight overlapping of single samples, which is consistent with the findings in Figs 6 and 7. Furthermore, for data under invisible conditions, there is a larger overlapping area in the ResNet model, while the features learned by LCNN have better separability.

### Results on MFPT bearing fault dataset

The MFPT bearing fault dataset provided by the American Society for Mechanical Failure Prevention Technology is a widely used open dataset in the current mechanical fault diagnosis field and has representative significance for mechanical vibration signal fault diagnosis. The dataset includes normal (Nor) data from the bearing test bench, outer race (OR) fault data and inner race (IR) fault data under different loads, and three real fault cases.

**Fig 10:**
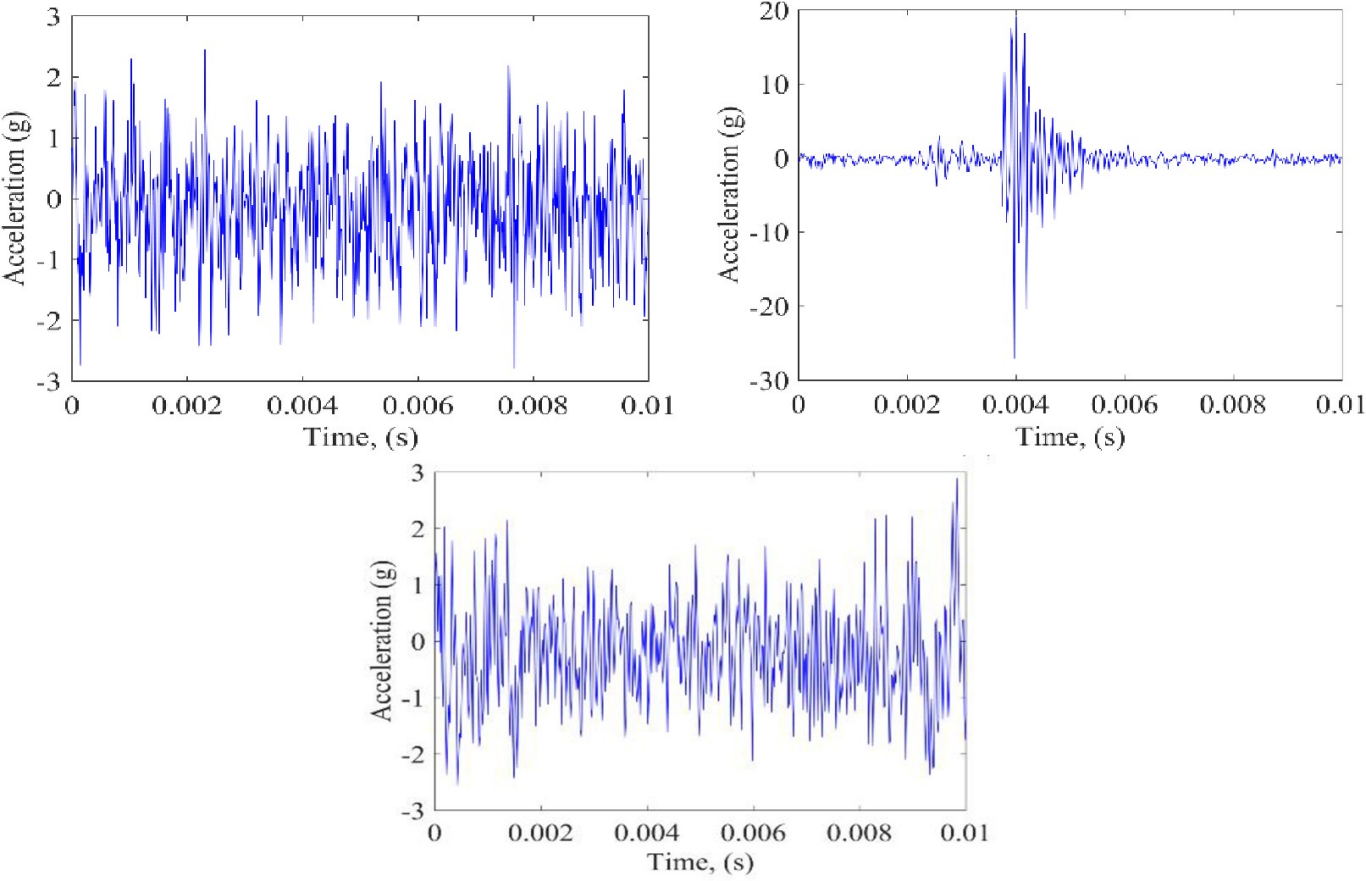
The MFPT bearing fault signals.

In order to ensure the sample balance, the training samples are constructed under 3 Nor conditions, 7 IR conditions, and 7 OR conditions, with the aim to verify the LCNN model proposed in this paper. Among them, Nor is 757,808 data points, IR is 1,025,388 data points, OR is 1,025,388 data points, and the data sampling rate is 97656. The number of picture waveforms of Nor, IR, and OR are waveforms 1800, 2100, and 2100, and the time step in each waveform diagram is 0.01 seconds, as shown in Fig 10.

During the experiment, 80% failure samples are used for training (70% for training and 10% for validation), and 20% for model testing. The recognition results of different models are shown in Table 5. It can be seen from Table 5 that LCNN-6 has the highest recognition accuracy, followed by LCNN-5 and ResNet, while LCCN-3 model has the lowest performance in accuracy, especially in the OR fault recognition. When trained directly, the LeNet and AlexNet models demonstrate poor recognition effect, the AlexNet model in particular as a result of vanishing gradient. Therefore, transfer learning is adopted to help models achieve better results in mechanical fault diagnosis. The other performance indicators of the model are the same as in 4.1.2, so they will not be described.

**Table 5:** The recognition results of models.

Model	Fault type			Overall accuracy(%)
	Nor(%)	IR(%)	OR(%)	
LCNN-3	100.00	90.95	65.00	77.86
LCNN-4	100.00	97.61	94.05	97.22
LCNN-5	100.00	99.76	100.00	99.92
LCNN-6	100.00	100.00	100.00	100.00
LeNet	100.00	97.14	98.09	98.41
AlexNet	100.00	99.05	100.00	99.68
ResNet	100.00	99.52	100.00	99.84
DBN	100.00	90.71	84.04	91.61
SAE	100.00	96.43	87.85	94.76
SVM	100.00	90.47	86.90	92.46

**Fig 11:**
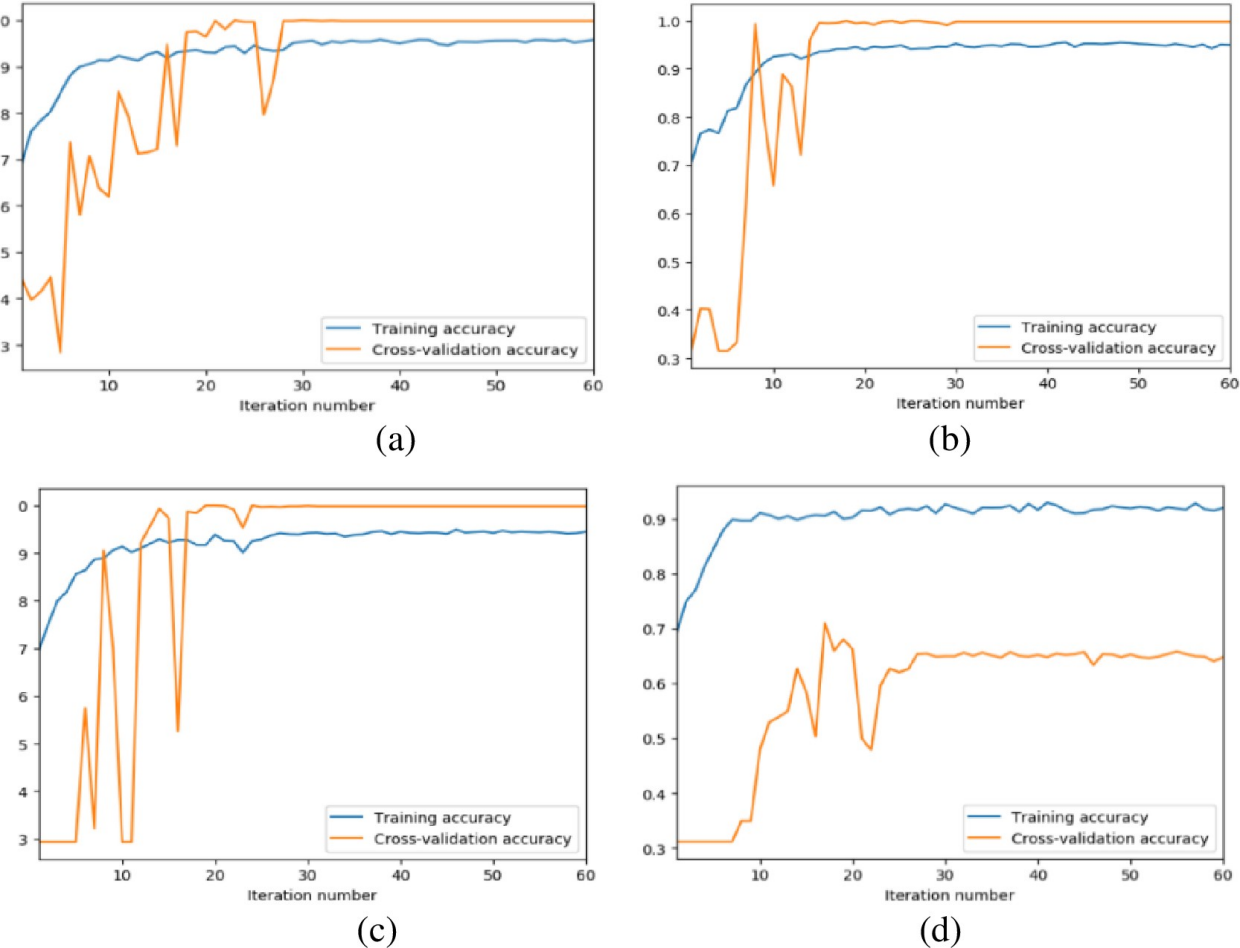
Training process curves of four LCNN models. (a) 6 blocks. (b) 5 blocks. (c) 4 blocks. (d) 3 blocks.

**Fig 12:**
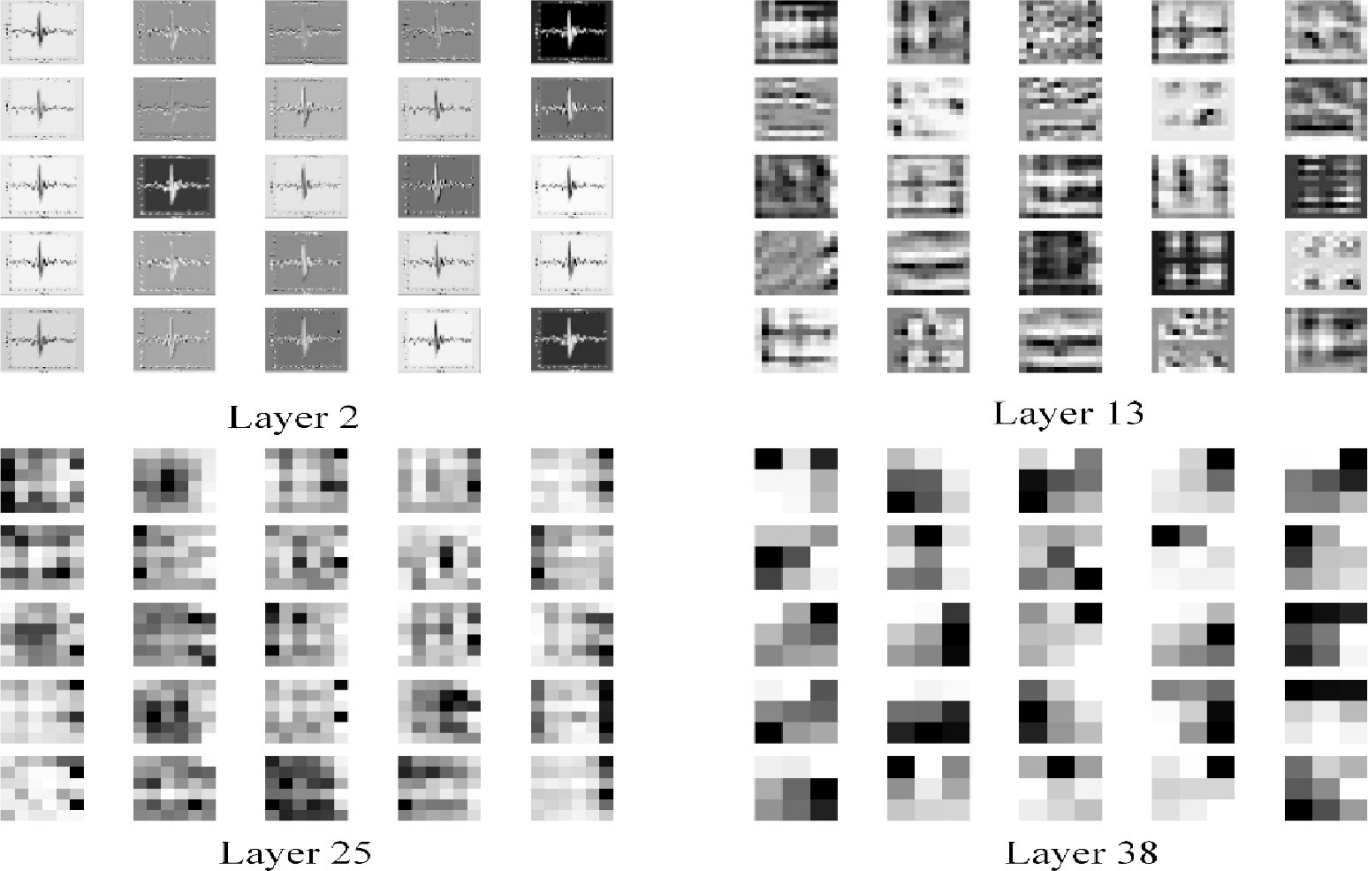
Visualization results of convolution features of the LCNN models. (a) Layer 2. (b) Layer 13. (c) Layer 25. (d) Layer 38.

**Fig 13:**
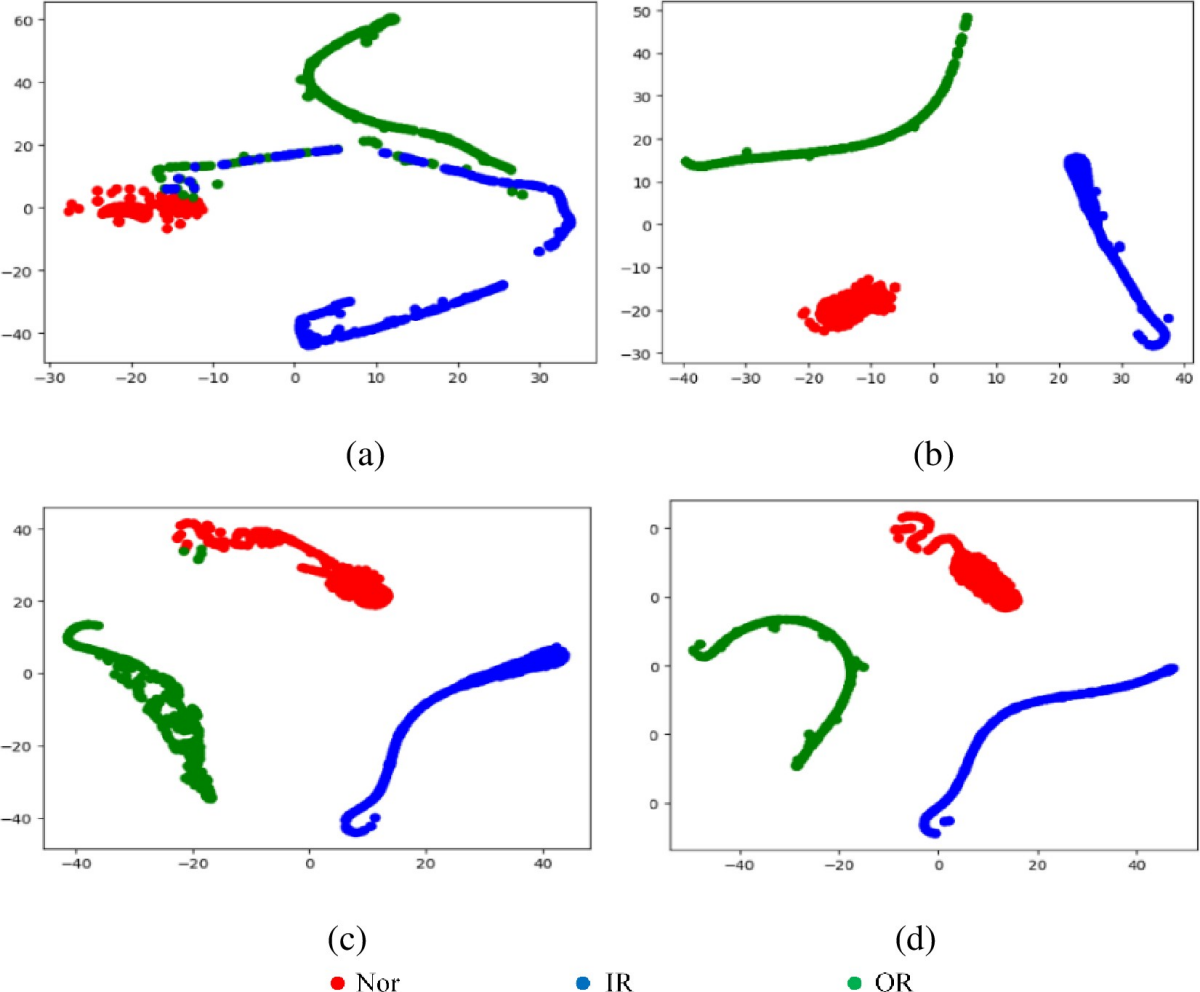
Visualization results of t-SNE. (a) LeNet. (b) AlexNet. (c) ResNet. (d) LCNN.

The training process of the 4 LCNN models constructed in this paper is shown in Fig 11. As the block number increases, the LCNN models become more and more robust. Besides, the more the block number, the smaller the fluctuation of the training and verification accuracy of the LCNN models, which indicates the better robustness of the models. Also, with the block number increasing, it takes fewer iterations for the models to stabilize, thus reducing training time. Figs 12 and 13 demonstrate the visualization results of the models’ convolution feature and t-SNE results. From the figures, the classification boundary of the LCNN models is obvious, and the classification areas occupied by the three fault types are far apart, further verifying the excellent performance of the proposed models on the accuracy index.

## Conclusion

In order to improve the fault diagnosis accuracy, and reduce the model parameters and storage for deployment, this paper proposes an LCNN-based intelligent fault diagnosis method for rotating machinery. Instead of traditional convolution operations, lightweight convolution blocks are used to construct LCNN models for accurate, automatic, and robust fault diagnosis of rotating machinery. Tensorboard and t-SNE are used to visualize the entire network. The superiority of the proposed method is also verified on the Case Western Reserve University Bearing fault dataset and the MFPT bearing fault dataset. The main conclusions are as follows:

1. The block number is in a proportion to the model accuracy. When the number increases to 6, the accuracy has reached more than 99.9% and become stable with the number further increasing. But the substantial increase in the parameter amount makes it difficult to train and deploy the model.
2. With the block number increasing, the model training curve becomes more robust, which indicates the model robustness. Meanwhile, it takes fewer iterations for the model to be stable, thus reducing the training time.
3. Compared with traditional methods, the LCNN model proposed in this paper has the highest diagnostic accuracy, with significantly less parameter amount and storage space. Thus, the proposed model is conducive to the deployment.
4. The entire network is visualized through Tensorboard and t-SNE. Initially, the proposed model mainly extracts signal contours, and then becomes abstract. The smoothness of the feature map shows that the automatic feature extraction in this paper is sufficient. The fault boundary of the fully connected layer is clearly separable, which proves the feasibility of the proposed method.

In the future, we plan to start with optimization methods to automatically reduce the model’s parameters and storage while automatically exploring the optimal LCNN architecture for fault diagnosis of rotating machinery. Considering the demands for real-time and fast data processing in the IIoT context, the processing speed of the model needs further improving.
